# Cutting-Edge Approaches in Respiratory and Critical Care Medicine

**DOI:** 10.3390/jpm13010105

**Published:** 2023-01-03

**Authors:** Ioannis Pantazopoulos, Ourania S. Kotsiou

**Affiliations:** 1 Department of Emergency Medicine, Faculty of Medicine, University of Thessaly, 41500 Larissa, Greece; 2 Department of Human Pathophysiology, Faculty of Nursing, University of Thessaly, 41110 Larissa, Greece

The COVID-19 pandemic has affected health care across the world, with respiratory and critical care medicine being affected the most. The response of clinicians and researchers who have provided care and research not only for patients with COVID-19, but in all areas of respiratory and critical care medicine in extraordinary circumstances has been impressive. In this Special issue of the *Journal of Personalized Medicine,* we invited scholars to contribute manuscripts that highlight and further the knowledge in the abovementioned challenging disciplines.

From the beginning of the pandemic, many nations introduced the use of face masks and respirators in the community to protect people against SARS-CoV-2 transmission. However, masks and respirators can provide different levels of protection depending on the type of the mask. Cloth masks provide the least protection, while surgical masks are safer and FFP/(K)N95 masks provide the highest protection [1]. On the other hand, prolonged mask use has been associated with a higher likelihood of a frequent cough, sputum production, dyspnea and panic attacks [1,2]. Given that the emergency phase of the pandemic is over, masks and respirators are recommended only for patients with a high mortality risk [3]. An increased mortality risk from COVID-19 has been observed for patients with the following factors: older age, male sex, β-thalassemia heterozygosity and respiratory disease [4]. Moreover, acute kidney injury, diabetes, hypertension, cardiovascular disease, cancer and obesity have also been reported as risk factors for a fatal outcome associated with SARS-CoV-2 [5].

The swift development of effective vaccines against COVID-19 was an unprecedented scientific achievement. In spite of this, the immunization of a critical proportion of the community proved to be very challenging mostly after the appearance of new strains of the virus that questioned the effectiveness of the vaccines and increased hesitancy. However, a large study from central Greece after the prevalence of new variants of the virus (Delta and Omicron) demonstrated that vaccination was still effective and provided high protection in terms of mortality and the clinical severity of COVID-19 [6]. Nevertheless, even in fully vaccinated patients, older age, higher viral load and a shorter period between symptom onset and hospital admission were associated with absence of anti-S SARS-CoV-2 antibodies upon hospital admission and poor clinical outcomes [7]. On the other hand, individuals vaccinated against COVID-19, but who were still infected by the virus, showed an “excellent boost” in their immune response [8].

For hospitalized patients, in addition to remdesivir, dexamethasone, immunomodulatory agents and monoclonal antibodies that have been approved for various severity stages of COVID-19, efforts for more largely available and safe drugs were continuous over the first two years of the pandemic. Among other methods, the administration of vitamin D was proposed mainly due to its immunomodulatory activity. However, no absolute conclusions could be drawn from a recent systematic review of the literature, due to the large variation in vitamin D supplementation schemes [9].

The incidence of pulmonary embolism (PE) has been reported to be around 2.6–8.9% in hospitalized COVID-19 patients, which is approximately nine-fold higher than in the general population [10]. Nevertheless, the prevalence of anticoagulant therapy-associated hemorrhagic complications in hospitalized patients with PE has been scarcely investigated. Pagkratis et al. retrospectively investigated the prevalence of hemorrhages in hospitalized PE patients during a 7-year period and found that one fifth of the patients hospitalized for PE suffered a non-fatal hemorrhage. The hemorrhages were mainly minor and lasted for 3 ± 2 days. Among low-molecular-weight heparins (LMWHs), nadroparin was related to a higher percentage of hemorrhages [11].

In the post-hospitalization period, pre-hospitalization methodical physical activity was associated with less dyspnea and a shorter recovery period, highlighting the importance of avoiding a sedentary life and engaging in exercise. On the other hand, in-hospital weight loss, comorbidities and dyspnea upon admission predicted a longer post-hospitalization recovery time [12].

The role of skeletal muscle mass in modulating immune response and supporting metabolic stress has been increasingly confirmed. Based on empirical data, patients with sarcopenia are speculated to have increased infection rates and poor prognoses amid the current COVID-19 pandemic [12]. In this context, this Special Issue aimed to shed light on the impact of less discussed comorbidities, such as the progressive loss of skeletal muscle mass and loss of muscle function, broadly known as sarcopenia in patients with chronic respiratory disease, such as bronchial asthma and/or COPD. Sarcopenia has been related to reduced lung function, a higher mortality risk, and higher risk of osteopenia and osteoporosis progression, leading to an increased risk of fractures, immobilization, and disability. Thus, physicians who examine sarcopenic patients with chronic airway diseases such as bronchial asthma or COPD should be able to appropriately collaborate with specialists who deal with nutrition and exercise, giving their patients a multimodal approach concerning these entities’ interplay and the optimum treatment [13].

The importance of exercise training was not only highlighted in COVID-19 patients. It is widely regarded as the cornerstone of pulmonary rehabilitation in patients with chronic airway diseases such as COPD. The COVID-19 pandemic has given telemedicine and telemonitoring a significant boost. Accordingly, the study of Barata et al. showed that the online pulmonary rehabilitation programs are not inferior to the traditional method in COPD patients [14]. Moreover, the use of rehabilitation programs for patients who have successfully completed anti-tuberculosis treatment has been highlighted as a potent multi-faceted measure in preventing the increase in mortality rates, as researchers concluded that a patient with a TB diagnosis, even after fully completing pharmacotherapy, is threatened by a potential life loss of 4 years, in comparison to healthy individuals [15]. Moreover, there is evidence that an eight-week course of a respiratory muscle training (RMT) program was helpful in increasing diaphragmatic thickness in COPD patients with an FEV1% of ≥ 30%, in addition to lung function and cognition [16].

Exercise itself may be classified as a fundamental therapeutic approach in that it restabilizes sleep architecture and quality. The sleep state has been associated with significant changes in respiratory physiology, including ventilatory responses to hypoxia and hypercapnia, upper airway and intercostal muscle tone, tidal volume and minute ventilation. In addition, sleep disruption may induce a pro-inflammatory state that is associated with an impairment of immune system function [17]. On the other hand, inspiratory muscle strength training (IMT) has shown promising results in managing both sleep apnea and arterial hypertension. The review of Papanikolaou et al. suggested that training inspiratory strength in athletes could prove to be beneficial in counteracting the detrimental effects of the aforementioned sleep disturbances [17]. Furthermore, a recent meta-analysis showed that mandibular advancement devices (MADs), instead of continuous positive airway pressure (CPAP), support the mandible in order to increase the airway space and reduce pharyngeal collapsibility [18].

An increase in particulate matter (PM2.5) levels due to environmental pollution has been associated with the increased incidence of COVID-19 and risk of mortality [19]. Moreover, an increase in PM2.5 levels above the daily limit has been significantly associated with an increase in emergency department visits due to exacerbation of asthma and COPD, upper respiratory tract infections and pneumonia [20]. Biomarkers are recognized as essential tools for the diagnosis and management of all the above-mentioned respiratory diseases. It has been previously suggested that suPAR, the soluble form of urokinase plasminogen activator receptor (uPAR), which is a glycosyl-phosphatidylinositol (GPI)-linked membrane protein, can be used as a marker of both inflammation and disease severity [21]. A recent study that investigated the effectiveness of suPAR as an indicator of the severity of asthma, a chronic inflammatory disease of the airways, demonstrated that suPAR levels could discriminate moderate uncontrolled asthma from severe asthma [22]. Its use was also studied in patients with pulmonary embolism, since suPAR is an integral part of the fibrinolytic system. However, its role is still unclear and needs further examination before definite conclusions can be drawn [23]. Interestingly, CRP, a traditional marker of inflammation, was identified as a predictor of 30-day survival and length of hospital stay in community-acquired pneumonia. Indeed, CRP with a cut-off point of 9 mg/dL on day 4 and 7 of hospitalization could predict survival with an area under the curve of 0.765 (0.538–0.992) and 0.784 (0.580–0.989), respectively. Moreover, a reduction in CRP above 50% by the fourth day of hospitalization could predict a shorter hospital stay [24]. Many biomarkers have also been studied in COPD patients. However, due to disease heterogeneity, especially at the level of COPD severity, progression, patients’ comorbidities and clinical status, there is a need for more personalized management. Specifically, the measurement and evaluation of each patient’s unique biomarker panel, rather than one unique biomarker, are expected in the coming years [25]. Furthermore, preliminary data from Ortakoylu et al. suggested the impressive performance of interferon (IFN)-gamma-inducible protein 10 (IP-10) as a marker to detect latent tuberculosis infection (LTBI) in patients with inflammatory rheumatic diseases (IRD). At the cut-off point of 2197 pg/mL, IP-10 showed 89% specificity with a sensitivity of 91% (AUC: 0.950; 95% CI 0.906–0.994) [26].

Nowadays, researchers warn that a tripledemic is heading our way this winter. This triple viral threat includes respiratory syncytial virus (RSV), influenza and COVID-19. All these viruses can cause cardiovascular manifestations, including arrhythmia, acute coronary syndrome, acute myocarditis, or acute heart failure, increasing cardiovascular morbidity and mortality. Researchers from Spain identified that high-sensitivity troponin T could predict mortality in influenza patients. In fact, patients with levels below 24 ng/L could be safely discharged from the emergency department, since at this cut-off point, high-sensitivity troponin T demonstrated both high sensitivity and a negative predictive value of 100% [27]. Future studies aimed at consolidating this result and examining its usefulness in patients with COVID-19 and RSV infection will be useful.

This Special Issue also presents significant scientific advances in non-COVID-19 critical care medicine. In a landmark article by Chalkias et al. who investigated the dynamic changes in determinants of venous return during hyperdynamic septic shock, the authors used two translational models (hemorrhagic and septic shock) to assess the decrease in stressed volume in severe septic conditions [28]. Most importantly, they identified for the first time the existence of another circulatory volume, the rest volume (Vr), that seems to have dual main functions in the steady state, i.e., to prevent an increase in venous resistance and maintain critical closing pressure. The maintenance of Vr may be a key factor for the cardiovascular stability reported during selective iloprost nebulization by Lee et al. [29], suggesting that Vr may have a key role in the prevention of V/Q mismatch. These conditions are important for maintaining (or improving) hemodynamic coherence, i.e., the translation of macrohemodynamics to effective cellular oxygenation, and thus a low endothelial inflammatory status. Inflammation and increased reactive oxygen species formation may affect all organ systems, including the lungs. Thus, the attenuation of NO exhalation by propofol and sevoflurane reported by Vekrakou et al. may imply the preservation of bronchial microcirculatory perfusion and decreased NO synthesis, due to the immunomodulatory effects and the effects on microcirculation mediated by anesthetics in steady states [30,31] and in disease [32,33]. All the aforementioned factors can improve heart–lung interactions and facilitate the application of lung-protective ventilation strategies, preventing injurious mechanical stretching of lung parenchyma, and subsequent progression to fibrosis in patients with ARDS [34].

In conclusion, the research findings provided in this Special Issue contributed to different areas of research by offering new knowledge and mapping out the research field of all areas of respiratory and critical care medicine.
